# Premenstrual disorders and risk of sick leave and unemployment: a prospective cohort study of 15 857 women in Sweden

**DOI:** 10.1136/bmjment-2025-301550

**Published:** 2025-07-07

**Authors:** Hang Yu, Yihui Yang, Elgeta Hysaj, Alicia Nevriana, Sara Hägg, Unnur Anna Valdimarsdóttir, Elizabeth Bertone-Johnson, Donghao Lu

**Affiliations:** 1 Unit of Integrative Epidemiology, Institute of Environmental Medicine, SE-171 77, Karolinska Institutet, Stockholm, Sweden; 2 Department of Medical Epidemiology and Biostatistics, SE-171 77, Karolinska Institutet, Stockholm, Sweden; 3 Center of Public Health Sciences, Faculty of Medicine, IS-101, University of Iceland, Reykjavik, Iceland; 4 Department of Biostatistics and Epidemiology, School of Public Health and Health Sciences, University of Massachusetts Amherst, Amherst, Massachusetts, USA; 5 Department of Health Promotion and Policy, School of Public Health and Health Sciences, University of Massachusetts Amherst, Amherst, Massachusetts, USA

**Keywords:** Adult psychiatry, Depression & mood disorders

## Abstract

**Background:**

Premenstrual disorders (PMDs) are prevalent and impair women’s quality of life, but their long-term influence on work capacity is unclear. Understanding the association between PMDs and subsequent sick leave and unemployment could inform interventions and policies.

**Objective:**

We hypothesised that women with PMDs have an increased risk of future sick leave and unemployment compared with those without PMDs.

**Methods:**

We conducted a prospective cohort study involving 15 857 women aged 15–60 years who were employed at baseline in the LifeGene Study, with linkage to population and health registers in Sweden. PMDs were identified from clinical diagnoses and symptom questionnaires; sick leave and unemployment data were obtained from national registers. Poisson regression estimated incidence rate ratios (IRRs) for sick leave and unemployment in women with versus without PMDs.

**Findings:**

A total of 2585 (16.3%) women (mean age 32.5 years) had probable PMDs. Over a median 9.17-year follow-up, 6741 (42.5%) and 1485 (9.4%) experienced at least one sick leave or unemployment, respectively. Compared with women without PMDs, those with PMDs had a 40% higher risk of sick leave (IRR 1.40, 95% CI 1.31 to 1.49) and a 27% higher risk of unemployment (IRR 1.27, 95% CI 1.10 to 1.46). Risk elevations were pronounced for sick leave≥90 days (IRR 1.69, 95% CI 1.50 to 1.91) and depression-related sick leave (IRR 1.41, 95% CI 1.27 to 1.56).

**Discussion:**

Women with PMDs are at increased risk of sick leave and unemployment, suggesting significant long-term socioeconomic burden associated with PMDs.

**Clinical implications:**

This study underscores the need for greater awareness of PMDs in clinical practice. Healthcare providers should recognise the potential impairment at work associated with PMDs, especially in women with recurrent symptoms or comorbidities like anxiety or depression.

**WHAT IS ALREADY KNOWN ON THIS TOPIC**

- Premenstrual disorders (PMDs) are common and can impair daily functioning, but their long-term impact on work-related outcomes, such as sick leave and unemployment, remains unclear.

**WHAT THIS STUDY ADDS**

- Women with PMDs face a significantly higher risk of future sick leave and unemployment compared with women without PMDs, particularly for long-term sick leave and depression-related leave.

**HOW THIS STUDY MIGHT AFFECT RESEARCH, PRACTICE OR POLICY**

- These findings indicate the need for intersectional efforts to mitigate the increased occupational and socioeconomic burden associated with PMDs.

## Background

 Premenstrual disorders (PMDs) include both premenstrual syndrome (PMS), which generally presents with milder symptoms and affects about 20–30% of reproductive-aged women, and the more severe premenstrual dysphoric disorder (PMDD), which impacts approximately 2.1–6.4%^1 2^ with debilitating psychological and physical symptoms typically emerging before menses.^2^ Since PMDD is characterised by more intense mood symptoms and functional impairment, while PMS symptoms are typically less severe, exploring these subtypes separately may help clarify how symptom severity influences women’s work lives. These symptoms recur with each menstrual cycle and significantly impair women’s quality of life throughout the reproductive years.^3^ Moreover, PMDs can have far-reaching effects on health. We have illustrated that women with PMDs face negative health consequences ranging from mental disorders, such as perinatal depression,^4^ suicidal attempt and death by suicide,^5^ to somatic conditions, such as early menopause and severe perimenopause symptoms,^6^ hypertension,^7^ cardiovascular diseases^5^ and autoimmune diseases (personal communication). Meanwhile, moderate-to-severe PMS as well as PMDD significantly increase the burden of healthcare expenditures for patients.^8^

Although many women with PMDs received their diagnosis in their 30s, a previous study by our group suggests that about 70% of them experience symptom onset before age 20.^9^ For many, PMDs may considerably affect women’s working life from early adulthood. PMDs have recently been associated with women’s ability to work, including decreased work performance and reduced work productivity.^10^,^12^ It is therefore plausible that PMDs are associated with more severe work impacts, such as increased rates of sick leave and unemployment, either due to PMDs themselves or related health conditions. Acute symptoms during the luteal phase, lasting up to a week, may directly lead to sick leave of a few days per cycle. However, the recurring nature of PMDs may gradually impair daily functioning, contributing to longer sick leave over time. Additionally, comorbidities such as depression and anxiety, along with other health impacts, may further affect work. Moreover, most women with PMDs have been reported to hide their symptoms from employers,^13^ which may delay support and exacerbate their condition, potentially leading to prolonged sick leave and higher unemployment risk.

More importantly, the cumulative effect of PMD symptoms, along with the negative emotions stemming from a lack of workplace support, significantly increases the likelihood of women with PMDs leaving their jobs by choice or via firing or layoff.^12^

However, only a few studies have investigated the relationship between PMDs and sick leave. A cross-sectional study showed that up to 16% of women with PMDs had sick leave during the past year.^14^ Another study from the USA reported that women with PMS experienced reduced productivity and increased absenteeism (OR 2.2; 95% CI 1.2 to 4.0) compared with women without, over a short follow-up of two menstrual cycles.^10^ While such short-term studies capture immediate symptom impacts, they are insufficient to observe outcomes requiring longer latency or diagnostic confirmation. For example, pregnancy-related complications, identified as one of the leading diagnoses for PMD-associated sick leave, typically emerge months after conception. Our study’s median follow-up of 9.17 years allows observation of these delayed and chronic consequences, which are inherently unmeasurable in studies with follow-up durations shorter than a full reproductive or career trajectory.

Moreover, we are not aware of any study investigating the influence of PMDs on unemployment. Together, we hypothesised that PMDs, given their recurrent and debilitating symptoms, would be associated with an increased risk of both sick leave and unemployment.

Our study aimed to examine whether women with PMDs have a higher risk of sick leave and unemployment compared with women without PMDs. Specifically, we investigated the overall risk of sick leave—including both short-term and long-term episodes—and evaluated whether this risk persists after accounting for psychiatric comorbidities. We also examined the association between PMDs and the 10 most common sick leave diagnoses and explored whether women with PMDs experience repeated sick leave episodes over time. Similarly, we assessed the risk of unemployment by determining if it is elevated among women with PMDs, if this association remains after adjusting for psychiatric conditions, and whether these women are more likely to experience recurrent episodes of unemployment.

Here, leveraging a prospective cohort of women employed at baseline in Sweden, we sought to evaluate these associations comprehensively. Specifically, we investigated the overall risk of sick leave, including both short-term and long-term sick leave, and whether this risk remains after accounting for psychiatric comorbidities, and examined the association between PMDs and the most common sick leave diagnoses.

## Methods

### Study design and population

We conducted a prospective cohort study of women of reproductive age contained within the LifeGene. LifeGene is a longitudinal population-based cohort in Sweden.^15^ Briefly, in 2009, residents in Sweden aged between 18 and 50 years were recruited through simple random sampling via internet survey, with subsequent snowball sampling and self-registration online. Participants provided data on the history of diseases and related risk factors (eg, lifestyle) through a baseline questionnaire and five follow-up questionnaires. The majority (66.6%) of participants completed the baseline questionnaire (ie, at recruitment) during 2009–2011. However, only the baseline questionnaire for assessment of covariates was used due to the high rate of loss to questionnaire follow-up.^15^

We linked women to multiple Swedish registers through their unique personal identification number. The Longitudinal Integration Database for Health Insurance and Labour Market (LISA) collects information on all employees’ education, income and occupation since 1990. The National Patient Register (NPR) contains data on all hospitalisation diagnoses since 1987 and specialist care-based outpatient diagnoses since 2001. The National Social Insurance Agency’s Micro Data for Analyses of the Social Insurance (MiDAS) records data on sick leave (longer than 14 days for all employees and shorter leaves for self-employees). Diagnoses in the MiDAS database are sourced directly from both primary and secondary care. The Swedish Prescribed Drug Register records filled prescriptions from all pharmacies in Sweden since July 2005. Primary care register from Stockholm County records clinical diagnoses made by general practitioners.

Among 24 332 female participants, we excluded women aged <15 or >60 years, and those without employment status at baseline according to LISA (online supplemental figure S1). Finally, 15 857 women were included in the analysis. Women were followed from the baseline until the first occurrence of sick leave/unemployment, death as identified from The National Cause of Death Register, emigration as obtained from the Total Population Register, age 67 (recommended age at retirement in Sweden) or 31 December 2020, whichever occurred first. The follow-up of unexposed women was additionally censored if they received a diagnosis of PMDs, and thereafter they contributed to the follow-up of the exposed group.

This study followed the STROBE (Strengthening the Reporting of Observational Studies in Epidemiology) reporting guidelines.

### Assessment of PMDs

PMDs were identified through healthcare registers capturing clinical diagnoses both prior to and during follow-up. However, as not all affected women seek medical care, we complemented registry data with baseline questionnaire assessments, which enable identification of women with substantial symptom burden who might otherwise remain undiagnosed. For the questionnaire assessments, we excluded women without menstrual cycles in the year prior to baseline, and those who missed at least 30% of items in PMDs assessment questionnaire. A participant was considered to have a PMD if indicated in either source.

As described elsewhere,^5^ in register data we identified clinical diagnoses of PMDs from the NPR (specialist care) and primary care register from Stockholm County, using International Classification of Diseases, 10th Revision (ICD-10) code (N943, 625E). To complement the diagnoses made in primary care in counties other than Stockholm, we also identified PMDs using prescriptions of serotonin reuptake inhibitors (SSRIs; Anatomical Therapeutic Chemical (ATC) code: N06AB) and other antidepressants (ATC code: N06AA, N06AB, N06AX) or hormonal contraceptives (ATC code: G03A, G02B) indicating a PMDs diagnosis or a specific dosing for PMDs (intermittent use of antidepressants) (online supplemental table S1).

In LifeGene, a modified version of the Premenstrual Symptoms Screening Tool (PSST) was used to assess PMDs at baseline. As described elsewhere,^16^ on confirming three screening questions about symptom timing and impact on psychosocial functioning, participants rated the severity of 15 physical, behavioural and emotional symptoms based on Diagnostic and Statistical Manual of Mental Disorders, Fifth Edition (DSM-V) criteria^2^ from 0 (none) to 3 (severe). Probable PMDs cases were categorised as follows: (1) one or more moderate/severe affective symptoms; (2) four or more other moderate/severe symptoms; and (3) at least one symptom with moderate/severe impact on relationships or social activities. Probable PMDs cases were further classified as PMS or probable PMDD; the latter was identified according to DSM-V^2^ criteria: (1) one or more severe affective symptoms; and (2) at least one symptom severely affecting relationships or social activities. The original PSST has been validated in other populations with a sensitivity of 90.9%.^17^

### Ascertainment of sick leave and unemployment

Information on sick leave was obtained from the MiDAS. Under the Swedish Sick Pay Act, employers are required to report to the National Social Insurance Agency when an employee has 15 days or more sick leave. Length of sick leave was obtained in MiDAS, and such information was used to classify short-term (less than 90 days) and long-term (90 days or more) sick leave, which is based on Swedish social insurance rules. After 90 days, a person’s ability to work is reassessed, and they may get extra support or rehabilitation. This cut-off helps separate shorter, routine absences from longer sick leaves that need more intervention. In addition, we obtained diagnoses underlying sick leave, based on certificates from the physicians for each sick leave episode.

Employment status was extracted from a LISA annual report variable, gainful employment status, based on (1) the presence of an income statement in November of the year in question, and (2) a minimum yearly income threshold that varies by year, age and sex, as estimated by Statistics Sweden using wage reports and labour market surveys. Self-employed individuals are considered employed regardless of their registered salary, as long as their company is recorded as active. The status was recorded as ‘employment’, ‘temporary employment’ and ‘unemployment’. The former two were categorised as ‘employment’ in this study.

### Covariates

Information on covariates was collected at baseline. Data on age, country of birth, civil status, parity, smoking, alcohol consumption, adverse childhood experiences (ACEs) including parental divorce, abuse by family member, non-sexual assault by someone known and sexual victimisation were collected through questionnaire, while body mass index (BMI) was calculated from self-reported weight and height. Since depression and anxiety are common comorbidities of PMDs,^1^ we obtained diagnoses of depression and anxiety from registers (online supplemental table S1). In addition, the education level at baseline was retrieved from LISA.

### Statistical analysis

#### Primary analyses

Poisson regression was used to estimate incidence rate ratios (IRR) with 95% CIs for sick leave and unemployment by comparing women with and without PMDs, respectively. IRR was also calculated for the PMDs subtypes (PMS and PMDD) based on questionnaire assessments. Two models were developed to address potential confounding factors, including demographics, socioeconomic status and lifestyle. In model 1, age was included as a fundamental confounder. In model 2, we additionally adjusted for BMI, civil status, ACEs, education level and country of birth, parity, smoking and alcohol consumption as potential confounders. Model 2 was selected as the primary model for analysis in this study.

To shed light on the severity and nature of sick leave, we estimated the association for short-term and long-term sick leave separately based on the National Social Insurance Agency’s definition; we also applied other cut-offs on the length of leaves to test the robustness of our findings. In addition, we evaluate the association for the ten most common diagnosis categories underlying sick leave.

#### Secondary analyses

We performed several additional analyses. First, PMDs are highly comorbid with depression/anxiety, which may explain our findings. We therefore performed analyses stratified by psychiatric comorbidities (depression/anxiety). Second, we also stratified the sick leave analysis by history of sick leave to illustrate the influence of previous sick leave.

#### Sensitivity analyses

Finally, although prospective symptom charting is recommended for PMDs diagnosis in the Swedish clinical guidelines, we lacked such information in registers. Therefore, we defined PMDs as those confirmed by both register-recorded diagnoses and questionnaire-based assessments, which likely improved the validity of PMDs identification. Since the percentage of missing values in the database we used is very small, we used Unknown as a categorisation of missing values. To avoid possible bias, we performed another sensitivity analysis after eliminating individuals with missing values.

The register data were prepared in SAS statistical software V.9.4 (SAS Institute, Cary, North Carolina, USA), while other data preparation and statistical analyses were performed in R software, V.4.0.5. Data were analysed from 18 January 2023 to 5 May 2024.

## Findings

At a mean age of 32.52 (Standard Deviation (SD) 8.45) years, 2585 (16.30%) women had either a register-recorded or questionnaire-assessed PMDs from baseline (n=1879) or reported during follow-up (n=706). Compared with women without PMDs, those with PMDs were older, more likely to report ACEs, and had a higher proportion of individuals born abroad. They were also more likely to have given birth and to have smoked in the past. Women with PMDs had a slightly higher mean BMI (all p<0.05; online supplemental table S2). Moreover, they were more likely to report a diagnosis of depression or anxiety.

### Risks of sick leave and unemployment

During a median follow-up of 9.17 years (mean age at end of follow-up 40.70 (SD 8.87) years), 6741 (42.51%) and 1485 (9.36%) women had sick leave and unemployment recorded in registers, respectively. Compared with unaffected women, women with PMDs had 40% (IRR 1.40, 95% CI 1.31 to 1.49) and 27% (IRR 1.27, 95% CI 1.10 to 1.46) higher risks of sick leave and unemployment, respectively (table 1, Model 2). Given that the questionnaire assessment was primarily designed to screen for PMDD, 1489 (57.60%) of all PMDs cases were classified as PMDD and 108 (4.18%) were identified as severe PMS. Statistically comparable associations for sick leave and unemployment were observed between PMDs and PMDD, though no association was found for severe PMS.

**Table 1:** IRRs and 95% CIs of unemployment and sick leave among women with PMDs compared with those without

	N of events (IR*)	Model 1†	Model 2‡
		IRR (95% CI)	IRR (95% CI)
Sick leave			
No PMDs	5623 (65.60)	1.00	1.00
PMDs	1118 (96.85)	1.45 (1.36 to 1.55)	1.40 (1.31 to 1.49)
PMS§	47 (70.95)	1.07 (0.80 to 1.42)	1.05 (0.79 to 1.40)
PMDD§	801 (94.97)	1.43 (1.33 to 1.54)	1.38 (1.28 to 1.49)
Unemployment			
No PMDs	1251 (11.58)	1.00	1.00
PMDs	234 (14.06)	1.33 (1.15 to 1.53)	1.27 (1.10 to 1.46)
PMS§	10 (11.39)	1.00 (0.54 to 1.87)	0.97 (0.52 to 1.81)
PMDD§	172 (14.45)	1.35 (1.15 to 1.58)	1.29 (1.10 to 1.52)

* Per 1000 person-years, unadjusted.

† Estimates were adjusted for age.

‡ Estimates were additionally adjusted for BMI, civil status, ACEs, education level and country of birth, parity, smoking and alcohol consumption.

§ In the analysis of PMDs subtype, only questionnaire-assessed PMDs were included because ICD code cannot be used to distinguish PMDD from PMS. Specifically, 1597 women with PMDs (108 PMS and 1489 PMDD) were included in the analyses for sick leave and unemployment.

ACEs, adverse childhood experiences; BMI, body mass index; ICD, International Classification of Diseases; IR, incidence rate; IRRs, incidence rate ratios; N, number; PMDD, premenstrual dysphoric disorder; PMDs, premenstrual disorders; PMS, premenstrual syndrome.

### Subtypes of sick leave

When analysing length of sick leave, a stronger association was suggested for long-term sick leave (defined as ≥90 days; IRR 1.69, 95% CI 1.50 to 1.91) than short-term sick leave (IRR 1.35, 95% CI 1.25 to 1.46) (table 2, Model 2). Similar patterns were observed when applying different cut-offs on the length of leave (online supplemental table S3). In the analysis of underlying causes of sick leave, the strongest association was noted for sick leave due to depression (IRR 1.41, 95% CI 1.27 to 1.56), post-traumatic stress disorder (PTSD) (IRR 1.40, 95% CI 1.28 to 1.54), followed by pregnancy complications (IRR 1.36, 95% CI 1.24 to 1.49) (table 3).

**Table 2:** IRRs and 95% CIs for short-term and long-term sick leave among women with PMDs compared with women without PMDs

	N of events (IR*)	Model 1†	Model 2‡
		IRR (95% CI)	IRR (95% CI)
Short-term§			
No PMDs	4142 (52.00)	1.00	1.00
PMDs	767 (73.77)	1.40 (1.29 to 1.51)	1.35 (1.25 to 1.46)
Long-term¶			
No PMDs	1481 (21.43)	1.00	1.00
PMDs	351 (39.43)	1.79 (1.59 to 2.01)	1.69 (1.50 to 1.91)

* Per 1000 person-years, unadjusted.

† Estimates were adjusted for age.

‡ Estimates were additionally adjusted for BMI, civil status, ACEs, education level and country of birth, parity, smoking and alcohol assumption.

§ Short-term: The days of sick leave were less than 90 days.

¶ Long-term: The days of sick leave were greater than or equal to 90 days.

BMI, body mass index; IR, incidence rate; IRRs, incidence rate ratios; N, number; PMDs, premenstrual disorders.

**Table 3:** IRRs with 95% CIs for the diagnoses underlying sick leave among women with PMDs compared with women without PMDs

Sick-leave diagnosis	N of events (IR*)	Model 1†	Model 2‡
		IRR (95% CI)	IRR (95% CI)
Post-traumatic stress disorder			
No PMDs	2962 (40.14)	1.00	1.00
PMDs	591 (61.27)	1.47 (1.34 to 1.60)	1.40 (1.28 to 1.54)
Pregnancy complications¶			
No PMDs	2947 (40.40)	1.00	1.00
PMDs	532 (57.71)	1.41 (1.29 to 1.55)	1.36 (1.24 to 1.49)
Depression			
No PMDs	2314 (33.09)	1.00	1.00
PMDs	454 (50.89)	1.48 (1.34 to 1.63)	1.41 (1.27 to 1.56)
Injury			
No PMDs	2236 (31.98)	1.00	1.00
PMDs	398 (45.16)	1.35 (1.21 to 1.50)	1.30 (1.17 to 1.45)
Anxiety			
No PMDs	2169 (31.15)	1.00	1.00
PMDs	401 (45.66)	1.41 (1.26 to 1.57)	1.34 (1.21 to 1.50)
Respiratory infections			
No PMDs	2125 (30.65)	1.00	1.00
PMDs	388 (44.42)	1.38 (1.24 to 1.54)	1.32 (1.18 to 1.47)
Malignant neoplasms of female genital organs§			
No PMDs	2050 (29.68)	1.00	1.00
PMDs	370 (42.67)	1.37 (1.22 to 1.53)	1.31 (1.17 to 1.47)
Uterine fibroids			
No PMDs	2027 (29.39)	1.00	1.00
PMDs	368 (42.31)	1.37 (1.22 to 1.53)	1.31 (1.17 to 1.47)
Sleep disorders			
No PMDs	2006 (29.13)	1.00	1.00
PMDs	361 (41.73)	1.37 (1.22 to 1.53)	1.31 (1.17 to 1.47)
Other			
No PMDs	2544 (35.69)	1.00	1.00
PMDs	459 (51.04)	1.38 (1.25 to 1.52)	1.32 (1.19 to 1.46)

* Per 1000 person-years, unadjusted.

† Estimates were adjusted for age.

‡ Estimates were additionally adjusted for BMI, civil status, ACEs, education level and country of birth, parity, smoking and alcohol consumption.

§ Malignancies of the breast and other female genital organs like the ovaries, uterus, cervix and vagina.

¶ Pregnancy complications: excessive vomiting in pregnancy, venous complications in pregnancy, antepartum haemorrhage, other pregnancy-related conditions.

ACEs, adverse childhood experiences; BMI, body mass index; IR, incidence rate; IRRs, incidence rate ratios; N, number; PMDs, premenstrual disorders.

### Additional analysis

We observed comparable associations for sick leave and unemployment, when comparing women with and without history of depression and anxiety (table 4). In addition, largely comparable results were yielded when analysing multiple events of unemployment/sick leave during follow-up (online supplemental table S4).

**Table 4:** IRRs with 95% CIs of sick leave and unemployment among women with PMDs compared with women without PMDs stratified by diagnosis of depression and anxiety separately

	Sick leave			Unemployment		
	N (IR*)	Model 1†IRR (95% CI)	Model 2‡IRR (95% CI)	N (IR*)	Model 1†IRR (95% CI)	Model 2‡IRR (95% CI)
Depression diagnosis						
Without						
No PMDs	4400 (58.37)	1	1	981 (10.61)	1	1
PMDs	635 (77.61)	1.32 (1.21 to 1.44)	1.29 (1.18 to 1.40)	143 (12.98)	1.3 (1.09 to 1.54)	1.23 (1.03 to 1.47)
With						
No PMDs	1223 (118.36)	1	1	270 (17.35)	1	1
PMDs	483 (143.67)	1.20 (1.08 to 1.34)	1.19 (1.07 to 1.33)	91 (16.19)	1.02 (0.80 to 1.30)	1.06 (0.82 to 1.36)
Anxiety diagnosis						
Without						
No PMDs	4602 (60.33)	1	1	1008 (10.68)	1	1
PMDs	781 (85.02)	1.39 (1.29 to 1.50)	1.35 (1.25 to 1.46)	164 (12.81)	1.32 (1.12 to 1.56)	1.27 (1.08 to 1.51)
With						
No PMDs	1021 (108.26)	1	1	243 (17.76)	1	1
PMDs	337 (142.96)	1.30 (1.15 to 1.47)	1.29 (1.13 to 1.46)	70 (18.23)	1.09 (0.83 to 1.42)	1.06 (0.80 to 1.40)

* Per 1000 person-years, unadjusted.

† Estimates were adjusted for age.

‡ Estimates were additionally adjusted for BMI, civil status, ACEs, education level, country of birth, parity, smoke, alcohol assumption.

ACEs, adverse childhood experiences; BMI, body mass index; IR, incidence rate; IRRs, incidence rate ratios; N, number of events; PMDs, premenstrual disorders.

We identified 411 (15.90%) women with PMDs confirmed by both register diagnosis and questionnaire assessment (online supplemental figure S1). Compared with the main analysis, the association was seemingly stronger for sick leave (IRR 1.97, 95% CI 1.63 to 2.40) whereas the association for unemployment (IRR 1.28, 95% CI 0.95 to 1.73) was similar but not statistically significant (online supplemental table S5).

## Discussion

### Summary of findings

In this prospective cohort study of 15 857 employed women with a maximum follow-up of 12 years, we found that women with PMDs, mainly PMDD, are at a higher risk of both sick leave and unemployment. Women with PMDs also have a greater risk increase for long-term sick leave and are more likely to take sick leave due to depression, PTSD and pregnancy complications. This study marks the first comprehensive assessment of the association of PMDs on sick leave and unemployment, providing important insights into the long-term health and socioeconomic impact of PMDs.

### Short versus long influence

Previous studies reported that PMDs affect work productivity and increase work absenteeism.^10^ However, many previous studies focused on specific occupations, which may have a limited generalisability to other occupations.^11^ Additionally, prior reports primarily comprised cross-sectional studies or prospective studies with short follow-up.^11^ The only prior study on sick leave^14^ was focused on short sick leave (less than 14 days), as such impact is typically confined within the week of late luteal phase.^18^ Indeed, in our data, no sick leave was directly prescribed for underlying diagnosis of PMDs (ICD-10 N94.3). However, the recurrent nature of PMDs^18^ may have a cumulative effect on work performance and the relationship with coworkers, which can contribute to adverse working-related outcomes in the long run. To our knowledge, this is the first study reporting that over a long follow-up period, women with PMDs had a 40% higher risk of being on sick leave for more than 2 weeks, compared with unaffected women. Moreover, our study has illustrated a stronger association with longer sick leave (90 days or more), lending support to the long-term impact on work capacity. However, future studies are warranted to illustrate the reasons underlying an association between PMDs and sick leave.

### Symptom severity

We found no elevated association of sick leave among women with PMS. The increased risk of sick leave might be related to the severity of premenstrual symptoms.^19^ Women with PMDD might struggle more with managing emotional symptoms, such as severe mood swings, anxiety or tension, which can interfere with their daily tasks, leading to frequent absences and decreased performance.^20^

### Influences of comorbidities and complications

Our findings may be explained by psychiatric comorbidities associated with PMDs. Indeed, we found women with PMDs were more likely to take sick leave due to depression and PTSD compared with unaffected women. It is well-documented that women with PMDs are likely to have psychiatric comorbidities, such as depression, anxiety and PTSD.^21 22^ Notably, our stratified analyses found that anxiety and depression, which are comorbidities of PMDs, do not explain the increased sick leave associated with PMDs. In addition, it has been shown that women with PMDs experience severe physical and psychological symptoms in early pregnancy,^23^ while our previous study found a higher risk of antepartum and postpartum depression among women with PMDs.^4^ In this study, we noted a positive association between PMDs and sick leave due to pregnancy complications. Future research is needed to understand PMD-associated pregnancy conditions and corresponding sick leaves. It is worth noting that we found a moderate difference in elevated association across underlying reasons for sick leave. This may be due to competing risk, that is, a patient may have multiple conditions/comorbidities, but only the condition leading to the first sick leave was accounted for.

### Unemployment

To our knowledge, no study has investigated the association of PMDs with unemployment. A cross-sectional study among Japanese nurses showed that symptoms during menstruation can increase willingness to quit a job.^24^ Here, we found that women with PMDD had a 30% higher risk of unemployment relative to unaffected women. Such risk can be attributed to the cumulative effect of recurrent PMDD episodes as well as the chronic comorbidities. It is important to note that in our study, we were unable to determine whether unemployment was due to voluntary termination or to being fired, which requires further investigation.

### Strengths and limitations

Our study is a prospective cohort study with sufficient length completeness of follow-up, including participants from the general population, and comprehensive adjustment for potential confounders. However, several limitations should be considered.

First, the probable PMDs identified from questionnaire assessment were based on retrospective symptom reporting, which could introduce potential misclassification. However, since participants were not aware of their future working outcomes, such misclassification was likely non-differential and would have attenuated the results towards null. Indeed, a stronger association with sick leave was observed when restricting analyses to PMD cases confirmed by both clinical diagnosis and questionnaire assessment, assuming a better validity of diagnosis. Moreover, given the nature of our data sources, mild/moderate PMS was not captured in the present study. Our findings may not apply to the group with milder symptoms. Second, MiDAS did not include sick leave less than 15 days except for self-employed individuals. Thus, our results may not be generalised to sick leaves of fewer days. Third, compared with the Swedish general population, LifeGene participants are younger and have higher socioeconomic status.^25^ However, we have accounted for these potential selection forces in our analyses, and we found comparable PMDs^3^ and sick leave^26^ prevalence among the general population and LifeGene participants. Fourth, while we adjusted for key sociodemographic and clinical factors, unmeasured confounding, such as genetic influences, may fully explain the observed associations. For unemployment (RR=1.27), the calculated E-value was 1.86 (lower bound of 1.43), suggesting that an unmeasured confounder would need to have at least a moderate association (risk ratio ≥1.86) with both PMDs and unemployment to fully account for the observed association. Regarding sick leave (RR=1.40), the E-value was 2.15 (lower bound of 1.95), indicating an even stronger confounding association would be required to completely explain this observed relationship. Future research with more comprehensive control for confounders, for example, genetically informed studies, is therefore essential to rule out residual confounding. The fifth limitation lies in our approach to handling missing data. Most variables had low missing rates (0.38%–2.0%), except BMI (7.2%). We coded missing values as ‘unknown’, which could introduce bias if missingness relates to both exposure and outcome. However, sensitivity analyses (online supplemental table S6) showed a minimal impact on our results. In addition, we cannot exclude the possibility of reverse causation, namely PMDs can be the consequence of some chronic conditions and related sick leave. For instance, isolation and reduced activity levels due to sick leave can exacerbate mental health problems,^27^ and unemployment can lead to increased symptoms of somatisation, depression and anxiety.^28^ However, we have observed comparable results regardless of depression history. Finally, our results may not generalise to countries with different labour market and welfare policies.

### Clinical implications

Our study suggests that employed women with PMDs are at a higher risk of sick leave and unemployment. These findings highlight the substantial health and socioeconomic burden associated with PMDs. Healthcare providers should be aware of the potential impairment on daily functioning and work capacity associated with PMDs.

## Supplementary material

10.1136/bmjment-2025-301550 online supplemental file 1

## Data Availability

Data may be obtained from a third party and are not publicly available.
