# Supplementary material for: Premenstrual disorders and risk of sick leave and unemployment: a prospective cohort study of 15 857 women in Sweden
Source: BMJ Ment Health. 2025 Jul 7;28(1):e301550. doi: 10.1136/bmjment-2025-301550 (PMC12258349; doi:10.1136/bmjment-2025-301550)
Supplement: online supplemental file 1 [file bmjment-28-1-s001.docx]

**Supplementary**

**Figure S1. Flowchart**

**Table S1. Codes for identification of premenstrual disorders**

**Table S2. Characteristics of women with premenstrual disorder (PMDs) and without PMDs, N (%).**

**Table S3. Incidence Rate Ratios (IRRs) and 95% confidence intervals (CIs) for Short-Term and Long-Term Sick Leave among women with premenstrual disorder (PMDs) compared to women without PMDs with multiple cut-offs**

**Table S4. Incidence rate ratios (IRRs) with 95% confidence intervals (CIs) of recurring work-related outcomes among women with premenstrual disorders (PMDs) compared to women without PMDs.**

**Table S5. Incidence rate ratios (IRRs) with 95% confidence intervals (CIs) of sick leave and unemployment among women with premenstrual disorders (PMDs) confirmed by both clinical diagnosis and questionnaire assessment, compared to women without PMDs.**

**Table S6. Complete case analysis of Incidence Rate Ratios (IRRs) and 95% confidence intervals (CIs) of unemployment and sick leave among women with premenstrual disorders (PMDs) compared to those without.**

**Figure S1. Flowchart**

**
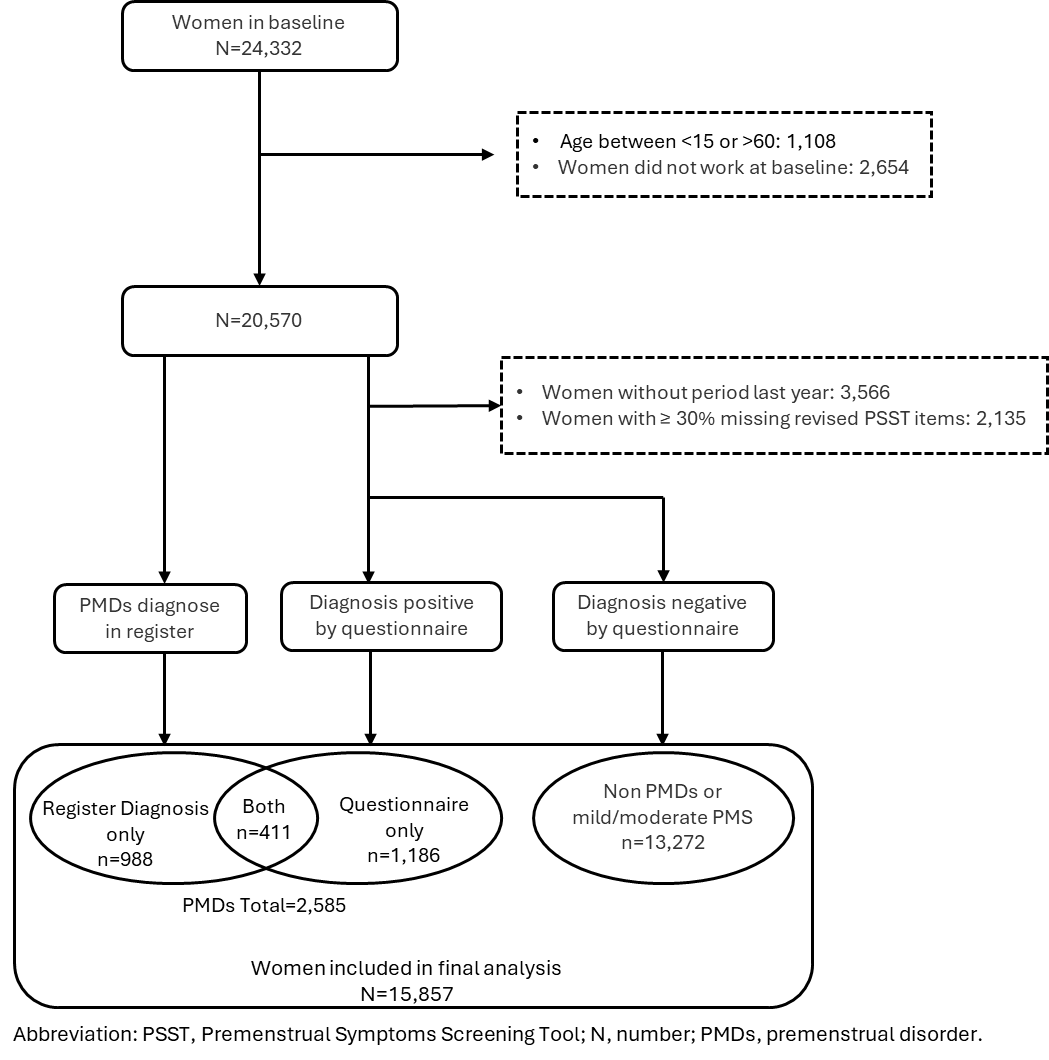
**

**Table S1. Codes for identification of premenstrual disorders**

| **Diagnosis** | **Source** | **Type** | **Codes** |
| --- | --- | --- | --- |
| Premenstrual disorders | NPR | ICD-10 | N943, 625E |
|  | SPDR | ATC | N06AA, N06AB, N06AX, G03A, G02B |
|  |  |  | With a written indication for PMDs in Swedish:  "PMS", "PREMENSTRUELLT SYNDROM", "PREMENSTRUELLT DYSFORSIKT SYNDROM", "PREMENSTRUELLT DYSFORI", "PMD", "PMDD", "PMDS", "MENS" |
| Depression | NPR | ICD-10 | F32-F33 |
|  | SPDR | ATC | N06A |
| Anxiety | NPR | ICD-10 | F40-F41 |
|  | SPDR | ATC | N05B |

Abbreviations: NPR, The National Patient Register; SPDR, The Swedish Prescribed Drug Register; ICD, International Classification of Diseases; ATC, Anatomical Therapeutic Chemical; PMDs, Premenstrual disorders.

**Table S2. Characteristics of women with premenstrual disorder (PMDs) and without PMDs, N (%).**

|  | No PMDs | PMDs | P |
| --- | --- | --- | --- |
|  | N=13,272 | N=2,585 |  |
| **Age (mean (SD))** | 32.37 (8.5) | 33.31 (8.1) | <0.001 |
| 15-19 | 367 (2.8) | 33 (1.3) | <0.001 |
| 20-29 | 5,511 (41.5) | 948 (36.7) |  |
| 30-39 | 4,339 (32.7) | 976 (37.8) |  |
| 40-49 | 2,652 (20.0) | 550 (21.3) |  |
| 50-60 | 403 (3.0) | 78 (3.0) |  |
| **BMI, kg/m2** |  |  | <0.001 |
| <18.5 | 420 (3.2) | 54 (2.1) |  |
| 18.5-25 | 9,939 (74.9) | 1,902 (73.6) | |
| 25.1-30 | 1,701 (12.8) | 319 (12.3) |  |
| >30 | 319 (2.4) | 59 (2.3) |  |
| Unknown | 893 (6.7) | 251 (9.7) |  |
| **Civil status** |  |  | <0.001 |
| Non-cohabitation | 5,187 (39.1) | 907 (35.1) |  |
| Cohabitation | 7,852 (59.2) | 1,588 (61.4) |  |
| Unknown | 233 (1.8) | 90 (3.5) |  |
| **ACEs** |  |  | <0.001 |
| No | 8,549 (64.4) | 1,423 (55.0) |  |
| Yes | 4,710 (35.5) | 1,088 (42.1) |  |
| Unknown | 13 (0.1) | 74 (2.9) |  |
| **Education Level** |  |  | 0.014 |
| Primary school | 603 (4.5) | 88 (3.4) |  |
| Secondary school | 3,390 (25.5) | 691 (26.7) |  |
| Post-secondary | 9,223 (69.5) | 1,801 (69.7) | |
| Other/Unknown | 56 (0.4) | 5 (0.2) |  |
| **Country of birth** |  |  | <0.001 |
| Sweden | 12,020 (90.6) | 2,279 (88.2) | |
| Not Sweden | 1,252 (9.4) | 306 (11.8) |  |
| **Parity** |  |  | <0.001 |
| 0 | 8,601 (64.8) | 1,510 (58.4) |  |
| 1-2 | 3,748 (28.2) | 806 (31.2) |  |
| 3 | 771 (5.8) | 188 (7.3) |  |
| Unknown | 152 (1.1) | 81 (3.1) |  |
| **Smoking status** |  |  | <0.001 |
| Never | 4,673 (35.2) | 656 (25.4) |  |
| Past | 7,522 (56.7) | 1,611 (62.3) |  |
| Current | 1,031 (7.8) | 244 (9.4) |  |
| Unknown | 46 (0.3) | 74 (2.9) |  |
| **Alcohol consumption** |  |  | <0.001 |
| Never | 410 (3.1) | 67 (2.6) |  |
| 1-3 times a month or less often | 6,963 (52.5) | 1,331 (51.5) |  |
| More than once a week | 5,685 (42.8) | 1,085 (42.0) |  |
| Unknown | 214 (1.6) | 102 (3.9) |  |
| **Depression^a^** | |  | <0.001 |
| No | 11,202 (84.4) | 1,728 (66.8) | |
| Yes | 2,070 (15.6) | 857 (33.2) |  |
| **Anxiety^a^** | |  | <0.001 |
| No | 11,392 (85.8) | 1,956 (75.7) | |
| Yes | 1,880 (14.2) | 629 (24.3) |  |
| **History of Sick leave** |  |  | <0.001 |
| No | 3,503 (56.8) | 763 (50.5) |  |
| Yes | 2,659 (43.2) | 747 (49.5) |  |

Abbreviation: BMI, body mass index (kg/m^2^); ACEs, adverse childhood experiences; N, number; %, percentage.

**^a^** depression and anxiety diagnosis from registers

Age was compared using one-way ANOVA. Categorical variables were compared using chi-square tests.

**Table S3. Incidence Rate Ratios (IRRs) and 95% confidence intervals (CIs) for Short-Term and Long-Term Sick Leave among women with premenstrual disorder (PMDs) compared to women without PMDs with multiple cut-offs**

|  | **N of events (IR ^a^)** | **Model 1^b^  IRR (95% CI)** | **Model 2^c^  IRR (95% CI)** |
| --- | --- | --- | --- |
| **Cut-off 30 days** | | | |
| Short-term ^d^ | | | |
| No PMDs | 1,986 (27.94) | 1.00 | 1.00 |
| PMDs | 346 (38.66) | 1.36 (1.21-1.52) | 1.33 (1.19-1.50) |
| Long-term ^e^ | | | |
| No PMDs | 3,637 (46.81) | 1.00 | 1.00 |
| PMDs | 772 (74.59) | 1.57 (1.45-1.69) | 1.49 (1.38-1.62) |
| **Cut-off 60 days** | | | |
| Short-term ^d^ | | | |
| No PMDs | 3,467 (45.04) | 1.00 | 1.00 |
| PMDs | 641 (64.18) | 1.40 (1.29-1.52) | 1.36 (1.25-1.49) |
| Long-term ^e^ | | | |
| No PMDs | 2,156 (30.03) | 1.00 | 1.00 |
| PMDs | 477 (51.23) | 1.67 (1.52-1.85) | 1.58 (1.43-1.75) |
| **Cut-off 90 days** | | | |
| Short-term ^d^ | | | |
| No PMDs | 4,142 (52) | 1.00 | 1.00 |
| PMDs | 767 (73.77) | 1.40 (1.29-1.51) | 1.35 (1.25-1.46) |
| Long-term ^e^ | | | |
| No PMDs | 1,481 (21.43) | 1.00 | 1.00 |
| PMDs | 351 (39.43) | 1.79 (1.59-2.01) | 1.69 (1.50-1.91) |
| **Cut-off 180 days** | | | |
| Short-term ^d^ | | | |
| No PMDs | 4,872 (59.01) | 1.00 | 1.00 |
| PMDs | 931 (85.03) | 1.42 (1.33-1.53) | 1.37 (1.28-1.48) |
| Long-term ^e^ | | | |
| No PMDs | 751 (11.34) | 1.00 | 1.00 |
| PMDs | 187 (22.39) | 1.88 (1.6-2.2) | 1.75 (1.49-2.07) |
| **Cut-off 365 days** | | | |
| Short-term ^d^ | | | |
| No PMDs | 5,352 (63.24) | 1.00 | 1.00 |
| PMDs | 1038 (91.76) | 1.43 (1.34-1.53) | 1.38 (1.29-1.48) |
| Long-term ^e^ | | | |
| No PMDs | 271 (4.23) | 1.00 | 1.00 |
| PMDs | 80 (10.02) | 2.23 (1.73-2.86) | 2.00 (1.54-2.6) |

Abbreviations: N, number; IR, incidence rate; IRR, Incidence rate ratios; CI, confidence interval; PMDs, premenstrual disorder.

^a^ Per 1000 person-years, unadjusted.

^b^ Estimates were adjusted for age.

^c^ Estimates were additionally adjusted for BMI, civil status, ACEs, education level, and country of birth, parity, smoking, and alcohol assumption.

^d^ Short-term: The days of sick leave were less than cut-off days.

^e^ Long-term: The days of sick leave were greater than or equal to cut-off days.

**Table S4. Incidence rate ratios (IRRs) with 95% confidence intervals (CIs) of recurring work-related outcomes among women with premenstrual disorders (PMDs) compared to women without PMDs.**

|  | **N (IR ^a^)** | | **Model 1^b^** | **Model 2^c^** |
| --- | --- | --- | --- | --- |
|  |  | | **IRR (95% CI)** | **IRR (95% CI)** |
| **Unemployment** | |  | | |
| No PMDs | 2,511 (17.84) | | 1.00 | 1.00 |
| PMDs | 507 (21.94) | | 1.32 (1.20-1.45) | 1.27 (1.15-1.4) |
| **Sick leave** | |  | | |
| **Total counts** |  | |  |  |
| No PMDs | 13,066 (92.83) | | 1.00 | 1.00 |
| PMDs | 2,838 (122.82) | | 1.3 (1.25-1.36) | 1.24 (1.19-1.29) |
| **Short-term counts^d^** |  | |  |  |
| No PMDs | 9,768 (69.4) | | 1.00 | 1.00 |
| PMDs | 1,998 (86.47) | | 1.23 (1.17-1.29) | 1.17 (1.11-1.23) |
| **Long-term counts^e^** |  | |  |  |
| No PMDs | 3,298 (23.43) | | 1.00 | 1.00 |
| PMDs | 840 (36.35) | | 1.52 (1.41-1.64) | 1.45 (1.34-1.57) |

Abbreviations: N, number of recurring work-related events; IR, incidence rate; IRR, Incidence rate ratios; CI, confidence interval; PMDs, premenstrual disorder.

^a^ Per 1000 person-years, unadjusted.

^b^ Estimates were adjusted for age.

^c^ Estimates were additionally adjusted for BMI, civil status, ACEs, education level, country of birth, parity, smoke, alcohol assumption.

^d^ Total number of episodes for sick leave was less than 90 days.

^e^ Total number of episodes for sick leave was greater than or equal to 90 days.

**Table S5. Incidence rate ratios (IRRs) with 95% confidence intervals (CIs) of sick leave and unemployment among women with premenstrual disorders (PMDs) confirmed by both clinical diagnosis and questionnaire assessment, compared to women without PMDs.**

|  | **N (IR ^a^)** | | **Model 1^b^** | **Model 2^c^** |
| --- | --- | --- | --- | --- |
|  |  | | **IRR (95% CI)** | **IRR (95% CI)** |
| **Sick leave** | |  | | |
| No PMDs | 5,623 (65.6) | | 1.00 | 1.00 |
| PMDs | 105 (137.96) | | 1.99 (1.64-2.41) | 1.97 (1.63-2.40) |
| **Unemployment** | |  | | |
| No PMDs | 1,251 (11.58) | | 1.00 | 1.00 |
| PMDs | 44 (13.36) | | 1.30 (0.96-1.76) | 1.28 (0.95-1.73) |

Abbreviations: N, number of events; IR, incidence rate; IRR, Incidence rate ratios; CI, confidence interval; PMDs, premenstrual disorder.

^a^ Per 1000 person-years, unadjusted.

^b^ Estimates were adjusted for age.

^c^ Estimates were additionally adjusted for BMI, civil status, ACEs, education level, country of birth, parity, smoke, alcohol assumption.

**Table S6. Complete case analysis of Incidence Rate Ratios (IRRs) and 95% confidence intervals (CIs) of unemployment and sick leave among women with premenstrual disorders (PMDs) compared to those without.**

|  | **N of events (IR^a^)** | **Model 1^b^** | **Model 2^c^** |
| --- | --- | --- | --- |
|  |  | **IRR (95% CI)** | **IRR (95% CI)** |
| **Sick Leave** |  |  |  |
| No PMDs | 5,080 (64.96) | 1.00 | 1.00 |
| PMDs | 959 (93.74) | 1.42 (1.33-1.53) | 1.39 (1.30-1.49) |
| -PMS^d^ | 42 (69.68) | 1.07 (0.79-1.45) | 1.07 (0.79-1.45) |
| -PMDD^d^ | 720 (93.19) | 1.42 (1.31-1.54) | 1.38 (1.27-1.49) |
| **Unemployment** |  |  |  |
| No PMDs | 1,105 (11.22) | 1.00 | 1.00 |
| PMDs | 199 (13.61) | 1.31 (1.12-1.52) | 1.27 (1.09-1.48) |
| -PMS^d^ | 8 (9.99) | 0.89 (0.44-1.78) | 0.86 (0.43-1.72) |
| -PMDD^d^ | 156 (14.39) | 1.38 (1.17-1.63) | 1.32 (1.12-1.57) |

Abbreviations: N, number; IR, incidence rate; IRR, Incidence rate ratios; CI, confidence interval; PMDs, premenstrual disorders; PMS, premenstrual syndrome; PMDD, premenstrual dysphoric disorder.

^a^ Per 1000 person-years, unadjusted.

^b^ Estimates were adjusted for age.

^c^ Estimates were additionally adjusted for BMI, civil status, ACEs, education level, and country of birth, parity, smoking, and alcohol assumption.

^d^ In the analysis of PMDs subtype, only questionnaire-assessed PMDs were included because ICD code cannot be used to distinguish PMDD from PMS. Specifically, 1,449 women with PMDs (97 PMS and 1,352 PMDD) were included in the analyses for sick leave and unemployment.
